# C-reactive protein is a predictive factor for complications after incisional hernia repair using a biological mesh

**DOI:** 10.1038/s41598-021-83663-6

**Published:** 2021-02-23

**Authors:** Julien Janet, Sophiane Derbal, Sylvaine Durand Fontanier, Stephane Bouvier, Niki Christou, Anne Fabre, Fabien Fredon, Thibaud Rivaille, Denis Valleix, Muriel Mathonnet, Abdelkader Taibi

**Affiliations:** 1 Visceral Surgery Department, Limoges University Hospital, Limoges, France; 2 University Limoges, CNRS, XLIM, UMR 7252, 87000 Limoges, France

**Keywords:** Biomarkers, Signs and symptoms

## Abstract

The introduction of biological or absorbable synthetic meshes has provided an alternative to conventional repair for incisional hernia. The ability to predict the development of complications after hernia surgery is important, as it guides surgical planning and patient management. This retrospective study assessed whether the postoperative C-reactive protein (CRP) level can predict complications after incisional hernia repair using biological mesh reinforcement. Patients who underwent incisional hernia repair surgery using biological meshes between February 2009 and February 2015 were screened for study inclusion. Patients included in the study were divided into two groups: those with and without postoperative complications. The two groups were analysed based on sex, surgical operation, length of intensive care unit stay (ICU), complications and mortality. Laboratory values, including white blood cell (WBC) count and CRP levels, were determined preoperatively and up to postoperative day (POD) 10. Postoperative complications requiring further management occurred in 32 of the 60 patients (53.3%). Among 47 patients, the mean CRP and WBC levels were 6.6 mg/L and 9.073 G/L in the group without complications vs. 141.0 mg/L, 16.704 G/L in the group with complications (p < 0.001). Patients with complications also had a longer ICU stay (10.1 vs. 0.6 days, p < 0.0001). A cut-off was 101 mg/L and offered 80.00% sensitivity (IC 61.43% to 92.29) and 95.24% specificity (76.18% to 99.88%) for postoperative complication. The rate of postoperative complications before POD10 was 95% in the group with CRP > 100 mg/L vs. 46% in the group with CRP < 100 mg/L (p = 0.000372). A high postoperative CRP level (> 100 mg/L) up to POD10 may serve as a predictor of postoperative complications in patients undergoing incisional hernia using biological meshes.

## Introduction

Incisional hernias occur when the surgical wound does not heal completely and are a common and significant complication after anterior abdominal wall incisions, such as midline or subcostal laparotomy^1–3^. Incisional hernia repairs are common surgical procedures and there are several techniques for restoring the integrity of the abdominal wall^4–7^. One of the most common methods is the use of a biological mesh such as Permacol. Among its benefits is that it can be used in septic settings, where synthetic meshes are unsuitable^8^. Early detection and management of complications following incisional hernia repair are crucial to reducing morbidity and mortality.

The C-reactive protein (CRP) is a well-known predictive factor for complications. The CRP level is a well-established indicator of postoperative complications, particularly following oesophageal, pancreatic and colorectal surgery^9–12^. Its ability to predict infectious complications, including after ventral hernia repair with synthetic mesh reinforcement has been reported recently^13^.

We aimed to study whether it is so in incisional ventral hernia repairs, and then determine what would be the best predictive cut-off. As a mesh infection is rare, we focused on a cohort of patients ‘at-risk’ for complications. Such patients were operated on in our hospital with a biologic mesh.

## Materials and methods

### Study population

- *Inclusion criteria* After informed consentment, all consecutive incisional hernias treated with the Permacol biologic mesh**,** at our university hospital between February 2009 and February 2015 were screened for inclusion in this retrospective study. Biological meshes were used in septic contexts in which synthetic materials are unsuitable.
- *Exclusion criteria* were a lack of surgical information and those where C-Reactive protein was lacking.
- *The following data* were recorded: age, sex, body mass index, smoking habit, diabetes, previous abdominal wall hernia, American Society of Anaesthesiologists score, hernia characteristics (location, width, length, primary and recurrent hernia), surgical characteristics (open or laparoscopic, operating time, emergency surgery, oncologic context, intestinal resection), mesh placement technique (onlay, inlay, sublay, underlay, or intraperitoneal placement), Altmeier wound classification (clean/clean contaminated/contaminated/dirty) and Ventral Hernia Working Group (VHWG) septic status.

### Definition of complications

Patients were assessed for complications, including infectious prosthetic complications, according to the Clavien-Dindo classification^15^. Recurrence of incisional hernia was also assessed. Recurrence was defined clinically as a perceptible gap in the abdominal wall with or without visceral bulging, or radiologically based on a computed tomography (CT) scan. Follow-up consisted of a phone interview and clinical examination in order ton analyse the rate of recurrence.

### Groups comparison according to CRP and WBC values

C-reactive protein was studied until postoperative day (POD)10 for all patients.

- We compared 2 subgroups (with or without complications) depending on their postoperative CRP and WBC level before POD10.
- A CRP cut-off value was used to define two others groups: those with a high or low postoperative CRP level, in order to analyse the rate of complications in each of these 2 groups.

Secondary endpoint was length of intensive care unit (ICU) stay.

### Ethics committee

This study was approved by the Ethics Committee of our hospital and registered under research registry. The paper has been reported in line with the STROCSS criteria^14^. All methods were carried out in accordance with French guidelines. This study was approved by the research ethics committees of Dupuytren Hospital Limoges and all research was performed in accordance with relevant guidelines and regulations.

### Statistical analyses

Qualitative variables are expressed as frequency distribution percentages, and quantitative variables are given as means. The categorical variables of the two groups were compared using a chi-square or Fisher’s exact test. The cut-off value for the CRP ratio was determined using receiver operating characteristic (ROC) curves. Sensitivity and specificity were calculated. A p-value < 0.05 was considered to indicate statistical significance. Statistical analyses were performed using Prism Graph.

### Ethics approval

This study was approved by the Ethics Committee of our hospital N° n°373–2020-2, and registered under research registry N°5606.

## Results

### Baseline patient characteristics and comparison of complication statuses

Among the 105 patients retrospectively screened for study inclusion, 13 were excluded due to other uses of a biological mesh (9 for fistula repair using biological mesh interposition, 2 for prophylactic reinforcement, 1 for hiatus hernia repair and 1 for strangulated umbilical hernia repair). In addition, 32 patients were excluded for a lack of information regarding the surgical procedure or the postoperative complications. Thus, the final study population consisted of 60 patients with incisional hernias who were treated with the biological mesh between February 2009 and February 2015. The characteristics of these patients are summarised in Table 1.

**Table 1:** Patients characteristics (*n* = 60).

	Population (*n*), mean (min–max)	Proportion (%)
**Sex**		
Male	27	45.0
Female	33	55.0
**Age**	65.8 (28–91)	
**BMI**	30.14 (17.1–41.4)	
**Comorbidities**		
Diabetes	13	21.7
Active smoking	13	21.7
**Laparotomy medical history**		
≤ 2	19	31.7
> 2	41	68.3
**ASA score**		
NA	1	1.6
I	3	5.0
II	34	56.7
III	18	30.0
IV	4	6.7
**Altemeier classification**		
1	25	41.7
2	10	16.7
3	17	28.3
4	8	13.3
**VHWG classification**		
1	2	3.3
2	23	38.3
3	22	36.7
4	13	21.7

Among the 60 patients, 32 (53.3%) developed complications. Table 2 lists the frequency of these complications, which included intra-abdominal abscesses, wound complications, abdominal wall abscesses, transit disorders and medical complications. There were no significant differences in the characteristics of the patients other than defect size, with respect to a particular complication (Table 3).

**Table 2:** Complications summary (n = 60).

**Complications**	n	Specific treatment
**Intra-abdominal abscesses**		
	3	Antibiotics
	1	Radiological drainage
	0	Surgical drainage
**Wound complications and abdominal wall abscesses**		
Disunion and sluices	6	3 surgical treatment 3 Vaccum Assisted Closure
Seroma	3	2 surgical drainage 1 radiological drainage
Abdominal wall abscesses	8	2 requiring no further treatment 2 drainage by nurse 3 surgery drainage 1 radiological drainage
**Transit disorder**		
Diarrhea	1	Medical treatment
Late transit recovery	1	Medical treatment
Occlusive syndrome	3	Surgery
**Medical complications**		
Anemia	3	Blood transfusion
Renal failure	3	Medical treatment
Heart Failure	2	
Respiratory failure	3	
Urinary Infection	1	
Pneumopathy	1	
Hyperkalemia	1	
Other 1: sigmoid and jejunal perforation 1: colon necrosis	2	Surgery
**Deaths**	4

**Table 3:** Patients characteristics depending on the complication status (n = 47).

	Patients without complications (n = 15) n (%) mean (min–max)	Patients with complications (n = 32) n (%) mean (min–max)	p-value
**Sex**			
Male	6 (40)	16 (50)	p = 0.55
Female	9 (60)	16 (50)	p = 0.55
**Age**	68.4 (45–88)	62.7 (28–91)	p = 0.14
**BMI**	40.0	30.5	p = 0.80
**Comorbidities**			
Diabetes	6 (40)	5 (15.6)	p = 0.13
Active smoking	3 (20)	8 (25)	p = 1
**Laparotomy medical history**			
≤ 2	3 (20)	11 (34.4)	p = 0.49
> 2	12 (80)	21 (65.6)	p = 0.49
**ASA score**			
NA	1 (6.7)	0 (0)	p = 0.32
I	1 (6.7)	2 (6.2)	p = 1
II	8 (53.3)	16 (50)	p = 1
III	5 (33.3)	10 (31.3)	p = 1
IV	0 (0)	4 (12.5)	p = 0.29
**Altemeier classification**			
1	8 (53.3)	9 (28.1)	p = 0.51
2	2 (13.3)	5 (15.6)	p = 1
3	4 (26.7)	11 (34.4)	p = 0.74
4	1 (6.7)	7 (21.9)	p = 0.40
**VHWG classification**			
1	1 (6.7)	0 (0)	p = 0.31
2	7 (46.7)	8 (25)	p = 0.18
3	5 (33.3)	13 (40.6)	p = 0.75
4	2 (13.3)	11 (34.4)	p = 0.17
**Carcinologic context**	7 (46.7)	9 (28.1)	p = 0.32
**Emergency context**	3 (20)	9 (28.1)	p = 0.72
**Associated digestive resection**	6 (40)	17 (53.1)	p = 0.76
**Defect size (cm**^**2**^**)**	50.9 (10.7–153.7) (n = 7)	108.6 (4.1–282.7) (n = 10)	p = 0.03
**Mesh size (cm**^**2**^**)**	465.7 (50–1000)	519.6 (150–2504)	p = 0.59
**Mesh placement technique**			
Onlay	1 (6.7)	0 (0)	p = 0.31
Inlay	2 (13.3)	11 (34.4)	p = 0.17
Sublay	1 (6.7)	1 (3.1)	p = 0.54
Underlay	0 (0)	0 (0)	p = 1
Intraperitoneal	11 (73.3)	19 (59.4)	p = 0.51
Other	0 (0)	1 (3.1)	p = 1

### Comparison of both groups (with or without complications) depending on their postoperative CRP and WBC level

Among the 60 patients, 13 patients were excluded for a lack of postoperative CRP measurement. As shown in Table 4, the CRP level was significantly higher in the group with than without complications: 141.0 vs. 62.6 mg/L (p < 0.001). Patients with complications also had a higher WBC count (16.7 vs. 9.1 G/L, p < 0.001) and a longer ICU stay (10.1 vs. 0.6 days, p < 0.0001). While the rate of recurrence was not statistically different between the two groups (p = 0.095), the recurrence rate tended to be higher in patients with complications when patients with missing data were excluded: 13 of 20 (65%) patients with complications vs. 2 of 8 (25%) patients without complications (Table 4).

**Table 4:** Comparison of the outcomes of patients depending on their complication status, (n = 47).

	Patients without complications (*n* = 15) *n *(%) mean (min–max)	Patients with complications (*n* = 32) n (%) mean (min–max)	p-value
**CRP levels** between POD7 and POD10 mean (min–max)	62.6 (4–210)	141.0 (1–359)	p = 0.000465
**Leukocyte levels** between POD7 and POD10 mean (min–max)	9.073 (4.60–12.200)	16.704 (4.500–48.000)	p = 0.0000901
**Intensive care unit stay** mean (min–max)	0.6 (0–4)	10.1 (0–36)	p = 0.0000322
**Recurrence**	2 (25)	13 (65)	p = 0.095

### The CRP cut-off analysis

**Figure 1:**
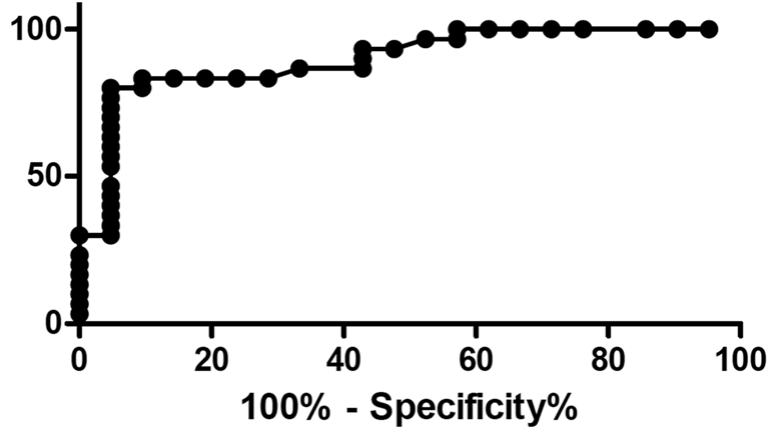
Receiver operating characteristic (ROC) curves for CRP. A cut-off was 101 mg/mL and offered 80.00% sensitivity (IC 61.43% to 92,29) and 95.24% specificity (76.18% to 99.88%) for postoperative complication, representing the optimal cut-off. The area under curve (AUC) was 0.89.

To determine the optimal cut-off for CRP, we analyzed the ROC curve (Fig. 1 and Annex 1). A cut-off was 101 mg/L and offered 80.00% sensitivity (IC 61.43% to 92.29) and 95.24% specificity (76.18% to 99.88%) for postoperative complication, representing the optimal cut-off. The area under curve (AUC) was 0.89.

### The rate of complications in each of group according to CRP value and CRP cut-off

The patients were divided into two groups depending on their postoperative CRP, based on a cut-off of 100 mg/L (< or > cut-off). The rate of complications was significantly higher in the high-CRP than in the low-CRP group (95.2% vs. 46.2%; odds ratio 0.0457; 95% confidence interval: 0.001–0.3699, p < 0.001). The difference in the severity of complications according to the Clavien-Dindo classification did not significantly differ between the two groups (Table 5).

**Table 5:** Comparison of the proportion of complications between groups, up to POD10 (CRP cut-off = 100), (n = 47).

	Patients with low PO level of CRP (*n* = *26*)	Patients with high PO level of CRP (*n* = *21*)	p-value
**Patients with complications**	12 (46.2)	20 (95.2)	p = 0.000372
**Patients without complications**	14 (53.8)	1 (4.8)	p = 0.000372
**Complications according to Clavien–Dindo**			
0	14 (53.8)	1 (4.8)	p = 0.000372
I	5 (19.2)	11 (52.4)	p = 0.029398
II	0 (0.0)	1 (4.8)	p = 0.446809
III	5 (19.2)	7 (33.3)	p = 0.325779
IV	0 (0.0)	0 (0.0)	p = 1
V	2 (7.7)	2 (9.5)	p = 1

## Discussion

Biological meshes consist of an organic biomaterial, typically porcine or bovine dermis, that has been decellularised to leave a collagen matrix that supports cellular colonisation, neovascularisation and progressive replacement with host tissue^16^. They have been used for many years to treat patients with peritonitis and abdominal sepsis, as they avoid the development of chronic infection necessitating mesh removal. Integration of the mesh into the host tissue creates a durable and permanent repair by inducing an inflammatory response that ultimately results in matrix remodelling^16^. However, biological meshes are more expensive than synthetic meshes and their use should therefore be restricted to the appropriate setting.

Early postoperative increases in the CRP levels of patients undergoing abdominal wall surgery have been reported and reflect a normal systemic inflammatory response. Therefore, in this study, to avoid confusion between a normal inflammatory response and the presence of a complication, the CRP level was measured until POD10 and the cut-off level was set at 101 mg/L.

CRP is synthesised by the liver and has a short half-life (~ 19 h), such that the serum level quickly returns to normal when the patient recovers. However, CRP has also been used as an early indicator of postoperative complications in abdominal surgery, in particular after oesophageal, pancreatic and colorectal surgery^9–12,17–21^. In this study, the ability of the CRP level to predict postoperative complications after incisional hernia repair surgery using a biological mesh was investigated. Sixty-six patients were included in this study, one of the largest series in which the ability of CRP to predict complications after incisional hernia repair was evaluated and the first in which the focus was on the use of biological mesh. Our results showed that not only the CRP level but also the WBC count predicted the development of postoperative complications after mesh-reinforced incisional hernia. The postoperative complication rate was high, 48.5%, and patients with a CRP level > 100 mg/L until POD10 had a much higher rate of complications (95%) than those with a CRP level below this cut-off (p < 0.001).

CRP is a powerful and early indicator of complications, as leakage after digestive resection and may thus be regularly monitored in digestive surgery patients^17^. Welsch et al. reported that CRP levels > 140 mg/dL on POD3 or 4 were predictive of infectious postoperative complications in rectal surgery patients^19^. Almeida et al. found that the same cut-off value, 140 mg/L on POD3, had a 78% sensitivity and 86% specificity in predicting anastomotic leakage in patients undergoing colorectal surgery^21^. According to Ortega-Deballon et al., patients with CRP values < 125 mg/L on POD4 after elective colorectal surgery can be safely discharged from the hospital^12^. Welsch et al. found that a CRP cut-off level of 140 mg/L on POD4 predicted inflammatory complications (pancreatic fistula or abscess) in pancreatic surgery patients, with a positive predictive value of 89.1%^10^. More recently, Mintziras et al. concluded that drain amylase associated to CRP could accurately predicted clinically relevant leakage after partial pancreaticoduodenectomy^22^.

In a study of patients who had undergone oesophageal resection, Deitmar et al. found that a CRP cut-off of 135 mg/L from day 2 onwards predicted leakage with an 80% sensitivity^11^.

In our study, patients with complications had a significantly higher CRP level than those without complications: 141.0 mg/L vs. 62.6 mg/L (p < 0.001). This suggests that CRP level is a predictor of complications in patients undergoing incisional hernia repair with a biological mesh. To our knowledge, only one study determined whether postoperative blood tests were valuable predictors of infectious complications after ventral hernia repair with mesh reinforcement^13^. In this study, Pochhammer et al. concluded that the postoperative CRP level had allowed the early prediction of the postoperative course, up to POD 7^13^. In that study the highest estimated CRP level occurred on POD5–6 and was 94.7 ± 77.6 in the group with complications versus 39.5 ± 29.1 in the group without complications (p < 0.001)^13^. In our retrospective study, the CRP cut-off value was 101 mg/L and based on these results, we have chosen this value to compare these both groups (with or without complications).

The difference in the WBC count between the two groups in our study was also significant: 16.7 vs. 9.1 G/L respectively (p < 0.001). WBCs are routinely measured based on CRP levels and changes in their dynamics over several days can provide further evidence of postoperative complications. However, in other studies the WBC count did not significantly improve diagnostic accuracy^23^.

Determining a CRP cut-off value for managing the postoperative course is not an easy task and usually involves compromises. When the CRP level is used to determine safe and early discharge, an assessment of the risk for complications is essential. In our study, a cut-off value of 100 mg/L revealed a significant difference between patients above and below the threshold, with complications rates of 95.2% vs. 46.2% respectively (p < 0.001). Given the high price of biological mesh, the increased risk of recurrence with every surgical revision and the frequent complexity of surgical revisions due to the patient’s surgical history, every patient with a postoperative CRP > 100 mg/L before POD10 should undergo a CT scan to look for complications.

### Limitations

Our study is limited by its retrospective and single-center design. One of the main limitations of our study was the late timing of the CRP measurement and the regularity of blood tests between the patients, as CRP values were assessed up to 10 days postoperatively. This time frame was chosen to avoid missing the frequent late complications after parietal surgery. In Pochhammer et al., the most frequent procedure-related complication was surgical site infection and it appeared after a median of 12 days postoperatively^13^. The median overall infectious complications occurred on POD 11 (2–89)^13^. Although this retrospective study have given interesting results, another limitation was the small sample size because, of the 105 patients screened, 45 were excluded, which could resulted in a loss of statistical power. Thus, a prospective study enrolling a large number of patients is needed to confirm the predictive value of a CRP cut-off of 100 mg/L during the first 10 postoperative days.

## Conclusion

This study examined the ability of CRP measurements during the first 10 postoperative days to predict complications in patients who underwent incisional hernia repair using a biological mesh. Patients with CRP values > 100 mg/L should be considered at high risk and should therefore undergo a CT scan to look for complications. Our findings may apply not only to biological meshes but also to other types of mesh used to repair complex, abdominal wall hernias.

## Supplementary Information

Supplementary Information.
