# Supplementary material for: C-reactive protein is a predictive factor for complications after incisional hernia repair using a biological mesh
Source: Sci Rep. 2021 Feb 23;11:4379. doi: 10.1038/s41598-021-83663-6 (PMC7902654; doi:10.1038/s41598-021-83663-6)
Supplement: Supplementary file 1 — Supplementary Information. [file 41598_2021_83663_MOESM1_ESM.pdf]

<u>Title:</u> C-reactive protein is a predictive factor for complications after incisional hernia repair using a biological mesh.

JANET Julien[1]; DERBAL Sophiane MD[1]; DURAND FONTANIER Sylvaine, MD[1-2]; BOUVIER Stephane MD[1]; CHRISTOU Niki MD, PhD[1]; FABRE Anne MD1; FREDON Fabien MD[1]; RIVAILLE Thibaud MD[1]; VALLEIX Denis, MD, PhD[1] ; MATHONNET Muriel, MD,PhD [1] ; TAIBI Abdelkader MD [1,2]

Cutoff	Sensitivity%	95% CI	Specificity%	95% CI	Likelihood ratio >
6.500	100,0	88,43% to 100,0%	4,762	0,1205% to 23,82%	1,05 >
20.50	100,0	88,43% to 100,0%	9,524	1,175% to 30,38%	1,11 >
33.50	100,0	88,43% to 100,0%	14,29	3,049% to 36,34%	1,17 >
37.00	100,0	88,43% to 100,0%	23,81	8,218% to 47,17%	1,31 >
39.50	100,0	88,43% to 100,0%	28,57	11,28% to 52,18%	1,40 >
41.50	100,0	88,43% to 100,0%	33,33	14,59% to 56,97%	1,50 >
45.50	100,0	88,43% to 100,0%	38,10	18,11% to 61,56%	1,62 >
49.50	100,0	88,43% to 100,0%	42,86	21,82% to 65,98%	1,75 >
51.50	96,67	82,78% to 99,92%	42,86	21,82% to 65,98%	1,69 >
53.00	96,67	82,78% to 99,92%	47,62	25,71% to 70,22%	1,85 >
54.50	93,33	77,93% to 99,18%	52,38	29,78% to 74,29%	1,96 >
58.00	93,33	77,93% to 99,18%	57,14	34,02% to 78,18%	2,18 >
61.50	90,00	73,47% to 97,89%	57,14	34,02% to 78,18%	2,10 >
66.50	86,67	69,28% to 96,24%	57,14	34,02% to 78,18%	2,02 >
71.50	86,67	69,28% to 96,24%	66,67	43,03% to 85,41%	2,60 >
72.50	83,33	65,28% to 94,36%	71,43	47,82% to 88,72%	2,92 >
74.00	83,33	65,28% to 94,36%	76,19	52,83% to 91,78%	3,50 >
76.00	83,33	65,28% to 94,36%	80,95	58,09% to 94,55%	4,38 >
79.50	83,33	65,28% to 94,36%	85,71	63,66% to 96,95%	5,83 >
85.00	83,33	65,28% to 94,36%	90,48	69,62% to 98,83%	8,75 >
91.00	80,00	61,43% to 92,29%	90,48	69,62% to 98,83%	8,40 >
101.5	80,00	61,43% to 92,29%	95,24	76,18% to 99,88%	16,80
> 111.0	76,67	57,72% to 90,07%	95,24	76,18% to 99,88%	16,10
> 118.5	73,33	54,11% to 87,72%	95,24	76,18% to 99,88%	15,40
> 127.0	70,00	50,60% to 85,27%	95,24	76,18% to 99,88%	14,70
> 133.0	66,67	47,19% to 82,71%	95,24	76,18% to 99,88%	14,00
> 141.0	63,33	43,86% to 80,07%	95,24	76,18% to 99,88%	13,30
> 150.0	60,00	40,60% to 77,34%	95,24	76,18% to 99,88%	12,60
> 156.5	56,67	37,43% to 74,54%	95,24	76,18% to 99,88%	11,90
> 159.5	53,33	34,33% to 71,66%	95,24	76,18% to 99,88%	11,20
> 170.0	46,67	28,34% to 65,67%	95,24	76,18% to 99,88%	9,80 >
185.5	43,33	25,46% to 62,57%	95,24	76,18% to 99,88%	9,10 >
193.0	40,00	22,66% to 59,40%	95,24	76,18% to 99,88%	8,40 >
198.5	36,67	19,93% to 56,14%	95,24	76,18% to 99,88%	7,70 >
204.5	33,33	17,29% to 52,81%	95,24	76,18% to 99,88%	7,00 >
208.5	30,00	14,73% to 49,40%	95,24	76,18% to 99,88%	6,30 >
219.0	30,00	14,73% to 49,40%	100,0	83,89% to 100,0%	>
233.5	23,33	9,934% to 42,28%	100,0	83,89% to 100,0%	>
243.5	20,00	7,713% to 38,57%	100,0	83,89% to 100,0%	>
250.5	16,67	5,642% to 34,72%	100,0	83,89% to 100,0%	>
261.5	13,33	3,755% to 30,72%	100,0	83,89% to 100,0%	>
293.0	10,00	2,112% to 26,53%	100,0	83,89% to 100,0%	>
322.0	6,667	0,8178% to 22,07%	100,0	83,89% to 100,0%	>
343.5	3,333	0,08436% to 17,22%	100,0	83,89% to 100,0%	
